# Epigenetic Signatures of Frailty: A Systematic Review, Meta-Analysis, and Network Analysis of the Chemical Exposome

**DOI:** 10.3390/ijms27135986

**Published:** 2026-07-03

**Authors:** Alejandro Eliu Cedillo-Rivero, Julian Daniel Rodriguez-Cuartas, Valentina Gomez-Zapata, Edgar Flores-Soto, Juan Carlos Gomez-Verjan, Nadia Alejandra Rivero-Segura

**Affiliations:** 1 Dirección de Investigación, Instituto Nacional de Geriatría, Mexico City 10200, Mexico; alejandroeliu@gmail.com (A.E.C.-R.); juliandaniel0716@ciencias.unam.mx (J.D.R.-C.); valentinagz111@gmail.com (V.G.-Z.); 2 Secretaría de Ciencias, Humanidades, Tecnología e Innovación (SECIHTI, Formerly CONAHCYT), Programa de Doctorado en Ciencias Biomédicas, Facultad de Medicina, Universidad Nacional Autónoma de México (UNAM), Mexico City 04510, Mexico; 3 Departamento de Farmacología, Facultad de Medicina, Universidad Nacional Autónoma de Mexico, Mexico City 04510, Mexico; edgarfloressoto@yahoo.com.mx

**Keywords:** frailty, epigenetics, DNA methylation, aging, chemical exposome, age-related disorders

## Abstract

Frailty is a multidimensional geriatric syndrome that lacks a consistent definition, complicating its clinical management. Epigenetic data suggest that frailty involves altered CpG sites, potentially driven by environmental epigenetic factors (the exposome) that influence aging. Systematically reviewing studies from 2009 to 2025, we quantified frailty prevalence, pooled weighted methylation beta values for associated CpG sites, performed enrichment analysis, and conducted structural network analysis to evaluate chemical interactions, following the PRISMA 2020 guidelines and with the study prospectively registered in PROSPERO (ID 1159037). Results showed a pooled frailty prevalence of 17.4% with extreme heterogeneity (I^2^ = 98.88%), and a combined methylated beta effect of −0.1378 (CI: −0.4156, 0.1400) with high heterogeneity (I^2^ = 100%), highlighting sources of variability. Interestingly, we found a CpG site (cg04772644) shared between Chinese and German cohorts, and, upon mapping, four frailty-related genes (CDC42BPB, SLC1A5, RXRB, and SLC22A18AS) were shared across cohorts. Indeed, these genes are significantly enriched in pathways including thrombin signaling, G protein-coupled receptor signaling, and immune cell differentiation signaling. Finally, our system toxicology analysis demonstrated that arsenite, bisphenol A, benzamide, dorsomorphin, and trichostatin A directly interact with the four shared genes, suggesting that the chemical exposome contributes to the observed epigenetic heterogeneity of frailty and the concomitant clinical manifestations.

## 1. Introduction

Frailty syndrome refers to a state of significant vulnerability to adverse outcomes [1]. This syndrome has a high prevalence in older adults and is characterized by a depleted physiological reserve across multiple biological systems [2]. Although the causes of frailty are related to environmental and behavioral factors, such as unhealthy lifestyles (e.g., physical inactivity or sedentarism, malnutrition, smoking, alcohol intake, and low socioeconomic status, among others), the molecular mechanisms orchestrating frailty syndrome remain incompletely understood. In this regard, several biological pathways have been implicated in the development of frailty, including systemic inflammation, mitochondrial dysfunction, neuroendocrinopathy, and epigenetic modifications [3]. Among these, epigenetic mechanisms—particularly DNA methylation (DNAm)—have emerged as promising contributors to the pathogenesis of frailty.

In particular, DNAm analysis has made enormous advances in understanding the epigenetic mechanisms of frailty. For instance, Gao and colleagues reported that methylation at previously identified smoking-related CpG (cytosine-phosphate-guanine dinucleotide) islands could serve as a prognostic biomarker of frailty, as assessed using the deficit-accumulation-based frailty index [4]. The data in this area corroborate a trend toward lower global DNAm in frail individuals, explained by reduced epigenetic control due to functional decline rather than chronological age during aging [5]. Still, some findings suggest that hypermethylation of specific CpG sites in the promoters of genes implicated in cell cycle regulation, apoptosis, metabolism, DNA repair, tumor-cell invasion, and cell signaling has been linked to these biological processes in the pathogenesis of age-related diseases [6]. This evidence suggests that environmental factors play an important role in the epigenetic component of this entity [2]. However, most existing epigenetic studies on frailty have been conducted in relatively homogeneous populations and have not adequately accounted for ethnic diversity, methodological differences across cohorts, or environmental influences.

In particular, the role of the chemical exposome—the cumulative exposure to environmental chemicals—remains largely unexplored in relation to frailty-associated epigenetic changes. Environmental factors such as air pollutants, endocrine disruptors, and heavy metals are known to alter DNA methylation patterns [7], yet their potential contribution to the observed heterogeneity in frailty-related epigenetic signatures has received little attention. Furthermore, epigenetic clocks, biomarkers that estimate a person’s biological age based on specific patterns of DNAm across the genome, also known as epigenetic age, have shown moderate associations with frailty, such as HannumAge (β = 0.06 [95% CI 0.02–0.09], I^2^ = 71.4%), PhenoAge (β = 0.07 [95% CI 0.03–0.11], I^2^ = 81.7%), and GrimAge (epigenetic clock related to death) (β = 0.11 [95% CI 0.06–0.15], I^2^ = 90.5%), suggesting that biological age acceleration may play a role in this syndrome and that such changes may be related to the environment [8].

Moreover, to address this gap, in the present study we conducted a systematic review and meta-analysis of frailty prevalence and DNA methylation patterns across multiple cohorts. We quantified the heterogeneity of epigenetic signals, identified CpG sites and genes consistently associated with frailty across populations, and performed network toxicology analyses to explore potential interactions between frailty-related genes and environmental chemicals. By integrating epigenetic and exposome data, this study aims to provide new insights into the biological mechanisms underlying frailty heterogeneity and its potential environmental determinants.

## 2. Materials and Methods

This systematic review and meta-analysis were conducted and reported in accordance with the Preferred Reporting Items for Systematic Reviews and Meta-Analyses (PRISMA) 2020 statement [9]. The protocol was prospectively registered in the International Prospective Register of Systematic Reviews (PROSPERO) under registration number 1159037. The completed PRISMA 2020 checklist is provided as a Supplementary Material (Table S1).

Gene symbols and names used throughout this study follow the official guidelines of the Human Genome Organization (HUGO) Gene Nomenclature Committee (HGNC). Gene symbols are written in italicized uppercase letters (e.g., SLC1A5), while the corresponding protein symbols are written in non-italicized uppercase letters.

**Figure 1:**
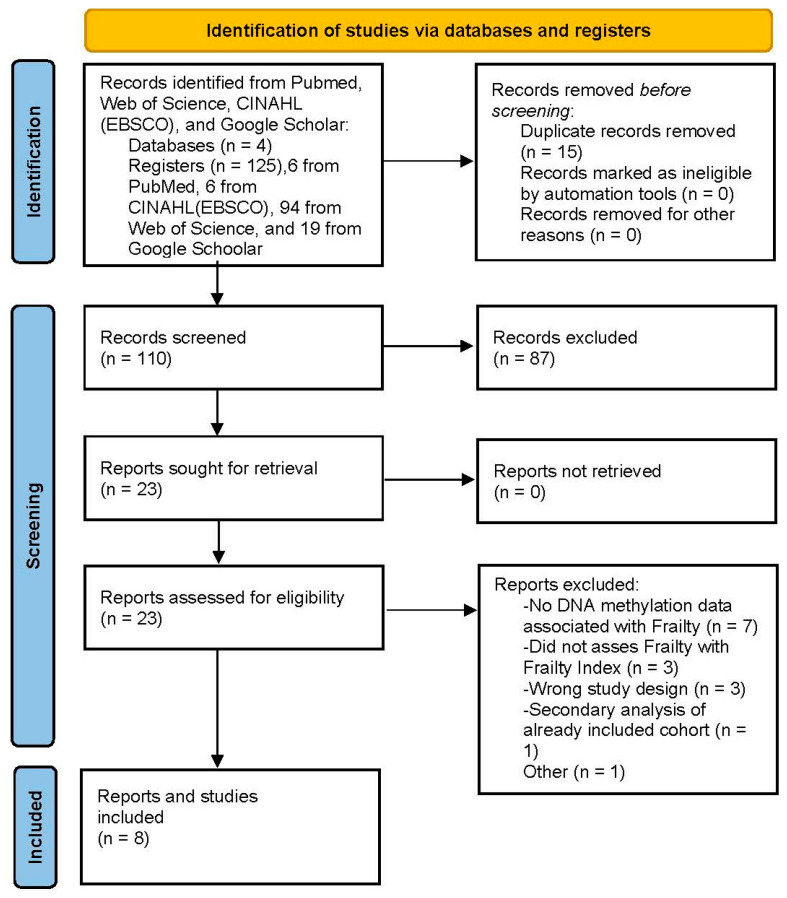
PRISMA 2020 flow diagram of study selection. The process involves three main steps: identification (articles retrieved from databases using MeSH terms), screening (articles excluded after their titles and abstracts are screened against the inclusion and exclusion criteria), and inclusion (the number of articles included for analysis). This study is registered with PROSPERO (ID 1159037).

According to our results depicted in Figure 1 we identified eight studies that met our inclusion/exclusion criteria. Using these studies, we performed all subsequent analyses. 

### 2.1. Search Strategy and Study Selection

This systematic review and meta-analysis were conducted and reported in accordance with the Preferred Reporting Items for Systematic Reviews and Meta-Analyses (PRISMA) 2020 statement [9]. The study protocol was prospectively registered in the International Prospective Register of Systematic Reviews (PROSPERO) under registration number 1159037. The completed PRISMA 2020 checklist is provided as a Supplementary Material (Table S1).

The initial search was conducted on 15 December 2025, and an update was performed on 12 May 2026, in four electronic databases using Medical Subject Headings (MeSH) terms: PubMed, CINAHL (via EBSCO), Web of Science, and Google Scholar. We included original studies published between 2014 and 2025 that reported epigenome-wide or site-specific DNA methylation data associated with frailty, as assessed by the frailty index or the deficit-accumulation approach. The search was limited to articles published from 2009 to 2025 in the Web of Science, PubMed, CINAHL, and Google Scholar databases. The following search strategies were used:

- PubMed: (“Frailty” [MeSH Terms]) AND “Epigenomics” [MeSH Terms] combined with age filters (Aged: 65+ years and 80 and over).
- CINAHL: Frailty AND epigenomics, with filters for English language and age (65+ years and 80 and over).
- Google Scholar: (Frailty) AND (DNA Methylation), with date range 2009–2025.
- Web of Science: (Frailty) AND (DNA Methylation), with date range 2009–2025, Document Types: Article, and Languages: English.

Equivalent strategies adapted to each database’s syntax were applied (full search strategies are provided in Supplementary Table S2). No language restrictions other than English were applied.

Eligibility criteria were defined using the Population, Intervention, Comparison, Outcome (PICO) framework:

- Population: Adults aged ≥60 years (community-dwelling or institutionalized).
- Intervention/Exposure: Studies reporting epigenome-wide or site-specific DNA methylation data (CpG sites and beta values).
- Comparison: Not applicable (observational studies).
- Outcome: Frailty assessed using the frailty index (FI) or deficit-accumulation approach, with statistically significant CpG sites associated with frailty (including *p*-values, false discovery rate (FDR), or beta values).

Exclusion criteria included: (1) studies not using the frailty index; (2) reviews, systematic reviews, meta-analyses, protocols, editorials, or conference abstracts; (3) studies without original DNA methylation data linked to frailty; (4) studies reporting only epigenetic clocks without individual CpG sites; (5) animal or in vitro studies; and (6) non-English publications.

Duplicate records were removed using Rayyan (v.1.6.1). Two independent reviewers (A.E.C.-R. and J.D.R.-C.) screened titles and abstracts, followed by full-text assessment. Disagreements were resolved by consensus or by a third reviewer (J.C.G.-V.).

### 2.2. Data Extraction

For each study, we extracted the following: first author, publication year, cohort name, sample size, number of CpG sites tested, and the number of CpG sites reported as significant. Data were extracted independently by two authors using a standardized template. The tables containing the data for this section are included in the Supplementary Materials (Tables S3 and S4).

### 2.3. Quality Assessment Analysis

The risk of bias in individual studies was assessed using the Joanna Briggs Institute Checklist for Prevalence Studies, which is appropriate for proportion-based studies [10]. This checklist evaluates whether the sample frame, data collection (recruitment), sample size, subject and setting description, data analysis, methodology, statistical analysis, and response rate are appropriate to accomplish the objectives of the study. According to the scoring system, the quality of the studies was evaluated as follows: high (7–9), moderate (4–6), and low (0–3). Data were visualized as a heatmap using the ggplot2 package (version 3.5.0) in RStudio (v.4.6.1). 

### 2.4. Meta-Analysis

Initially, a meta-analysis of proportions was conducted in R (version 4.4.2) using the metafor package (version 4.8-0) [11]. Proportions were transformed using the Freeman–Turkey double arcsine method. A random-effects model (DerSimonian and Laird) was used to account for anticipated heterogeneity across cohorts. Heterogeneity was assessed using the I^2^ and τ^2^ statistics. Sensitivity analyses were conducted by removing each study in turn. A Baujat plot was used to identify influential studies. Subgroup analyses and meta-regressions were performed to explore potential sources of heterogeneity. Then, we performed a meta-analysis of weighted methylated beta values using data from the list of CpGs extracted for each included cohort by inverse-variance weighting, in which the contribution of each cohort was weighted by the precision of its effect-size estimate, ensuring that studies with smaller standard errors had a proportional influence on the pooled estimate.

In the sensitivity analysis, we excluded the study by Li X et al. [12] because it exhibited the greatest heterogeneity. Both the random forest created in this section and a heatmap showing the number of significant CpGs associated with frailty and their directions are shown in the Supplementary Materials. 

### 2.5. Enrichment and Pathway Analysis

Genes extracted from the list of 108 CpG sites significantly associated with frailty across all included studies were mapped and enriched for Kyoto Encyclopedia of Genes and Genomes (KEGG) pathways (version 1.46.0) using the ReactomePA package (version 1.50.0) in R [13]. A Gene Ontology (GO) enrichment analysis was also conducted using the topGO package (10); we obtained GO terms for biological processes. The top 10 enriched pathways were identified with the topGO package (version 2.58.0) in R, and a hierarchical meta-functional analysis was performed to prioritize biological relevance. Only pathways surviving multiple-testing correction (*p*-value < 0.05) were visualized. The complete list of genes and CpGs is in Supplementary Materials, Table S4. Furthermore, a Gene Ontology enrichment analysis was performed using only the four genes shared across cohorts (*CDC42BPB*, *SLC1A5*, *RXRB*, and *SLC22A18AS*). The KEGG-enriched pathway analysis yielded no single pathway significantly associated with frailty.

### 2.6. Interactome Analysis of Frailty-Related Genes

Initially, we performed an interactome analysis of the gene list associated with the significant CpG site in frailty using the Comparative Toxicogenomics Database (CTD) (version updated in 2025) [14], accessed on 15 December 2025. The CTD generates an interactome by curating a search engine to extract and organize known relationships from the scientific literature. This process is based on two pillars: manual data curation and knowledge integration. The CTD analysis compiles each relationship, annotated with an interaction type (e.g., “*increases expression*,” “*decreases activity*,” “*causes*”). We used Cytoscape software (version 3.7) to analyze, reconstruct, and perform structural analyses of all networks obtained during the study [15,16].

### 2.7. Network Biology Analysis

We extracted the 100 most strongly associated compounds with frailty from the CTD. We use the CytoHubba plugin for Cytoscape to obtain a network of the most connected nodes using the Maximum Neighborhood Component (MCC) [16]. From the biological network, we extracted the top 30 genes and “toxicants” most strongly associated with frailty. From this list, we included all chemical compounds related to the top 20 genes and linked them to those genes. We elaborated a descriptive figure with the top 10 “toxicants” most associated with frailty syndrome. 

## 3. Results

### 3.1. Six of the Eight Studies Included in the Meta-Analysis Showed High-Quality Evidence

According to our results, studies from Zhang Y. (2018) [17], Li X. (2022) [12], Jiang M. (2025) [18], Gao X. (2017) [4], Gale CR. (2018) [19], and Collerton J. (2014) [20] are the studies with a high quality score since they met most of the items included in the Joanna Briggs Institute Checklist for Prevalence Studies (Supplementary Materials, Figure S1). This evidence aligns with studies that provide reliable data regarding the DNAm patterns in frailty.

### 3.2. Epigenetic Studies in Frailty Showed a Global Hypomethylation Pattern

**Figure 2:**
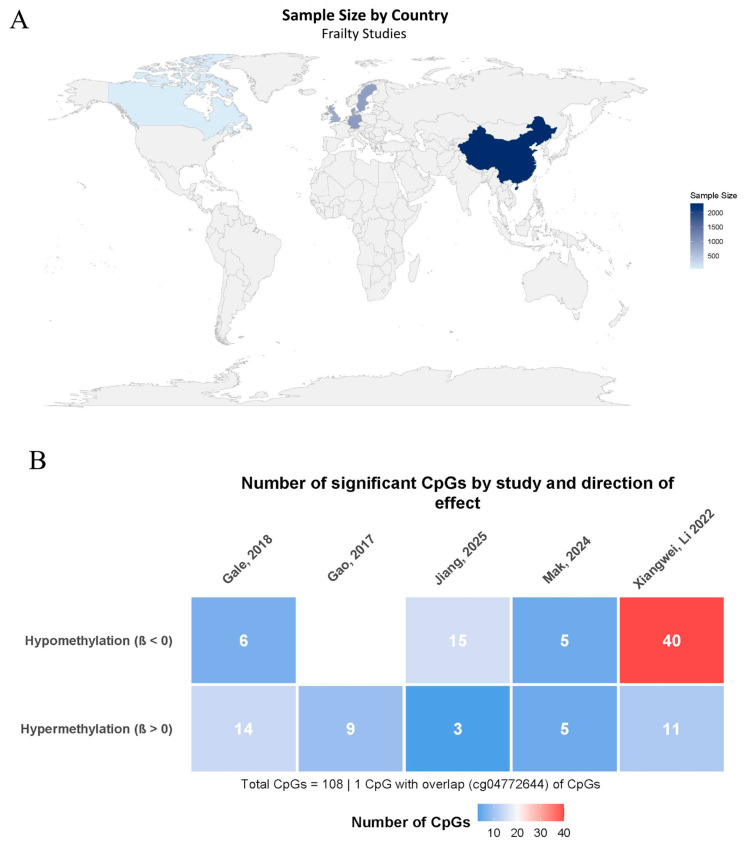
Geographical distribution and global methylation patterns of the studies included. (**A**) Map of the distribution of the studies included worldwide by sample size. (**B**) (refs. [4,12,18,19,22]). Heatmap showing the number of significant CpG sites and their direction of differential methylation for each study. This figure includes only five of the eight selected studies because they report the false discovery rate (FDR), *p*-value, and standard error (SE) for both hypo- and hypermethylated CpGs. Note that the five studies shown here are not the same subset used for the weighted beta meta-analysis (see Table 1).

According to the results from Figure 2A, the eight included studies were conducted in Canada (n = 56, Chadha, et al., 2023 [21]), the UK (Gale et al., 2018 [19], and Collerton et al., 2014 [20], respectively n = 791 and 321), Sweden and Denmark (n = 915, Mak et al., 2024 [22]), Germany (Zhang et al., 2018 [17], n = 2321; Li et al., 2022 [12], n = 3291; and Gao et al., 2017 [4], n = 978), and China (Jiang et al., 2025 [18], n = 21,654). Interestingly, despite large differences in sample sizes across cohorts, frail individuals exhibited a global hypomethylation pattern (66 sites hypomethylated vs. 42 sites hypermethylated), accounting for 61.11% of significant CpG sites (Figure 2B).

**Table 1:** Contribution of individual studies to each meta-analysis.

Reference	Prevalence Meta-Analysis (n = 8)	Weighted Beta Meta-Analysis (n = 5)	Sensitivity Analysis	Figure 2 Heatmap (Direction of Methylation)	Quality (Based on JBI Checklist for Prevalence Studies)
Zhang Y. (2018) [18]	Yes	No	Included	No	High
[12]	Yes	Yes	Excluded	Yes	High
[17]	Yes	Yes	Included	Yes	High
[4]	Yes	Yes	Included	Yes	High
Gale C.R. (2018) [19]	Yes	Yes	Included	Yes	High
Collerton J. (2014) [20]	Yes	No	Included	No	High
[21]	Yes	No	Included	No	Low
[22]	Yes	Yes	Included	Yes	Moderate

### 3.3. Frailty Studies Show Substantial Heterogeneity in Combined Prevalence and Weighted Methylated β Values

**Figure 3:**
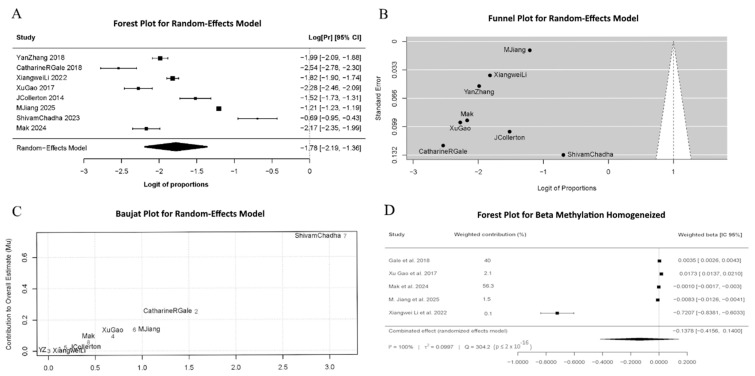
Overall results from a meta-analysis study [4,12,17,18,19,20,21,22]. (**A**) Forest plot. Comparison of proportional meta-analysis using randomized model. (**B**) Funnel plot. comparison of proportional meta-analysis with randomized model. (**C**) Baujat plot for the eight studies included. This plot clearly shows that the survey by Chadha et al. [21] makes the largest contribution to the estimated heterogeneity in prevalence across all cohorts. (**D**) Random forest of five studies. This plot compares weighted methylated beta across cohorts.

Using the meta-analysis of proportions methodology with the random-effects model, we found significant heterogeneity in prevalence estimates across the included studies (Figure 3). In Figure 3A, the combined prevalence observed across the analyzed studies is 17.4% (logit estimate = −1.7492, *p* < 0.0001), with substantial heterogeneity across cohorts, as evidenced by the I^2^ value of 98.88%, Tau^2^ of 0.5432 (SE = 0.3193), and Q statistic of 824.1857 (df = 7, *p* < 0.001), as shown in Figure 3B. Moreover, the Baujat plot (Figure 3C) shows that the study by Chadha et al. accounts for most of the heterogeneity in prevalence across cohorts.

Additionally, we performed a meta-analysis of weighted methylated beta values. Only five of the eight included studies provided the necessary data (CpG-level effect sizes with standard errors) to conduct inverse-variance weighted meta-analysis (Table 1). These five studies were included in the primary analysis, which showed a combined methylated beta effect of −0.1378 (95% CI: −0.4156 to 0.1400) with substantial heterogeneity (I^2^ = 100%, τ^2^ = 0.0997, Q = 304.2, df = 4, *p* < 2 × 10^−16^) (Figure 3D).

To evaluate the robustness of these findings and identify influential studies, we performed a sensitivity analysis by sequentially excluding each of the five studies. Exclusion of the study by Li Xiangwei et al. (2022) [12] produced the largest reduction in heterogeneity (Table 1). After removing this study, the combined effect was attenuated to 0.0029 (95% CI: −0.0075 to 0.0133), although heterogeneity remained high (I^2^ = 99.6%, τ^2^ = 0.0001, Q = 159, *p* < 2 × 10^−16^). These sensitivity analysis results are presented in Supplementary Figure S2. Table 1 summarizes each study’s contribution to the analyses conducted in this review.

Additionally, to obtain a more robust measure for the comparison, we performed a meta-analysis of weighted methylated β values using the list of CpGs associated with frailty. It is important to note that we included only the five studies depicted in Figure 1, as they contained the data required to perform the analysis described above. As a result, we found significant heterogeneity in the prevalence of frailty, with a combined methylated β effect of −0.1378 (CI: −0.4156 to 0.1400), heterogeneity I^2^ = 100%, Tau^2^ = 0.0997, and Q = 304.2 (df = 4, *p* < 2 × 10^−16^) (Figure 3D). 

In addition, we performed a sensitivity analysis, excluding the study by Li Xiangwei, as this study contributes to most of the heterogeneity presented; the results were consistent with maintaining a significant heterogeneity (combined methylated beta effect of 0.0029 (CI −0.0075, 0.0133), with heterogeneity I^2^ = 99.6%, Tau^2^ = 1 × 10^−4^, and Q = 159 (*p* < 2 × 10^−16^); these results are presented in the Supplementary Materials (Figure S2).

### 3.4. Epigenetic Alterations in Frailty Are Associated with the Immune System and Bone Morphogenesis

Once we obtained the prevalence of frailty and heterogeneity across studies, we aimed to assess the impact of differentially methylated CpGs on biological pathways. Thus, we retrieved a list of 108 CpG sites reported by the eight studies included in the meta-analysis. According to KEGG database results (Table 2, upper), thrombin signaling, G alpha signaling, and platelet activation, signaling, and aggregation are among the most altered pathways associated with the CpGs reported in frail individuals. In contrast, the TopGO (Table 2, lower) shows that T-cell differentiation, retinoic acid receptor signaling pathway, lymphocyte differentiation, and endochondral bone morphogenesis are the main biological processes associated with the 108 CpGs found in frail individuals. 

### 3.5. CpG Site cg04772644 Is Shared Between the Chinese and German Populations

**Figure 4:**
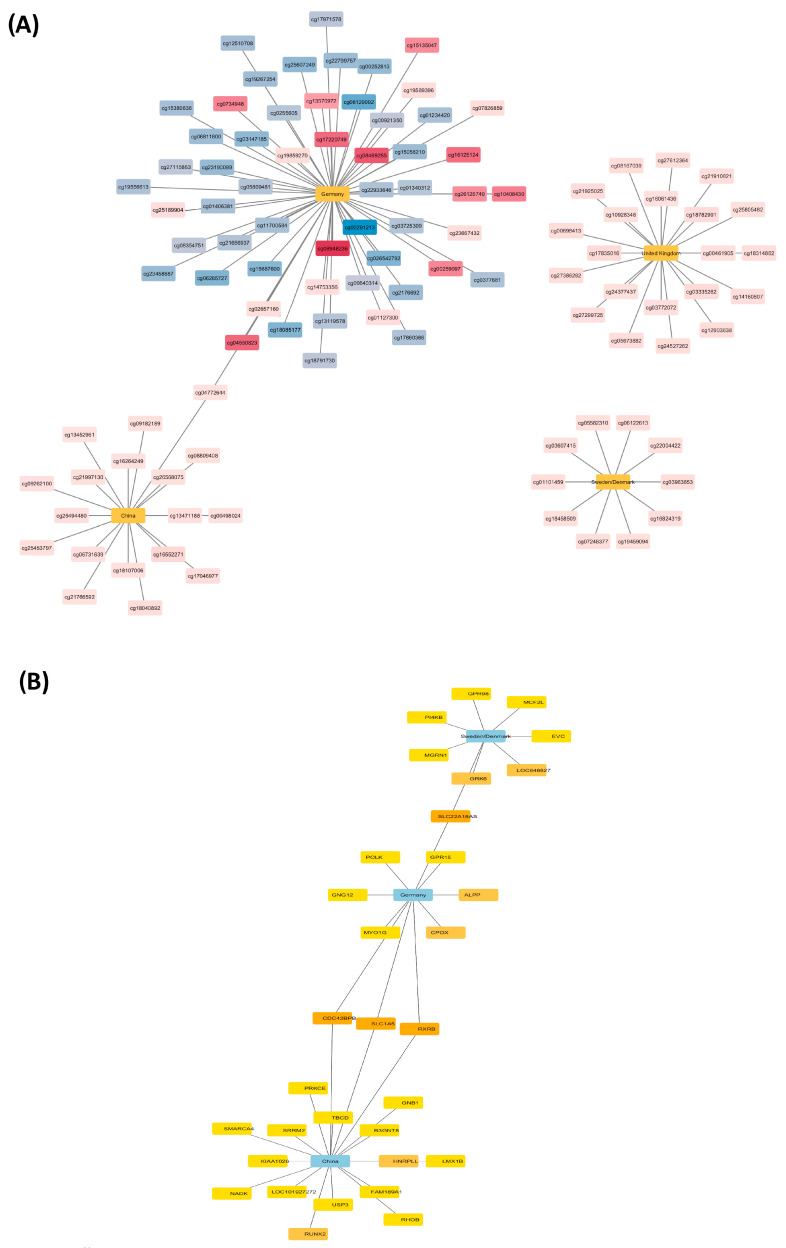
Structural network analysis of CpGs and studies. (**A**) In the network, CpG sites associated with frailty by population of origin are represented by yellow nodes; we observed every CpG site and gene on a scale from dark blue to dark red according to their beta-methylated value (dark blue to dark red nodes). (**B**) In this network, we plotted each CpG site gene associated with a yellow-to-orange node scale according to its beta-methylated value.

Moreover, to delve into frailty heterogeneity, we aim to identify whether, across populations, there are CpGs that share commonalities. As shown in Figure 4A, only one CpG site (cg04772644) was shared between the Chinese and German populations, while the remaining populations were unique to their respective CpGs.

### 3.6. Structural Network Analysis Reveals Four Frailty-Related Genes Across Cohorts Independently of Ethnicity

Then, to assess whether ethnicity affects the genes altered in frailty, we built the structural network shown in Figure 4B, which revealed that four frailty-related genes (CDC42BPB, SLC1A5, RXRB, and SLC22A18AS) are shared across the Chinese, German, and Danish/Swedish cohorts.

Interestingly, the functional enrichment analysis (Table 3) showed that these genes are involved in L-glutamine import across the plasma membrane, positive regulation of the vitamin D receptor signaling pathway, and glutamine transport, among others. 

### 3.7. Toxicants That Interact with the Most Frailty-Related Genes

**Figure 5:**
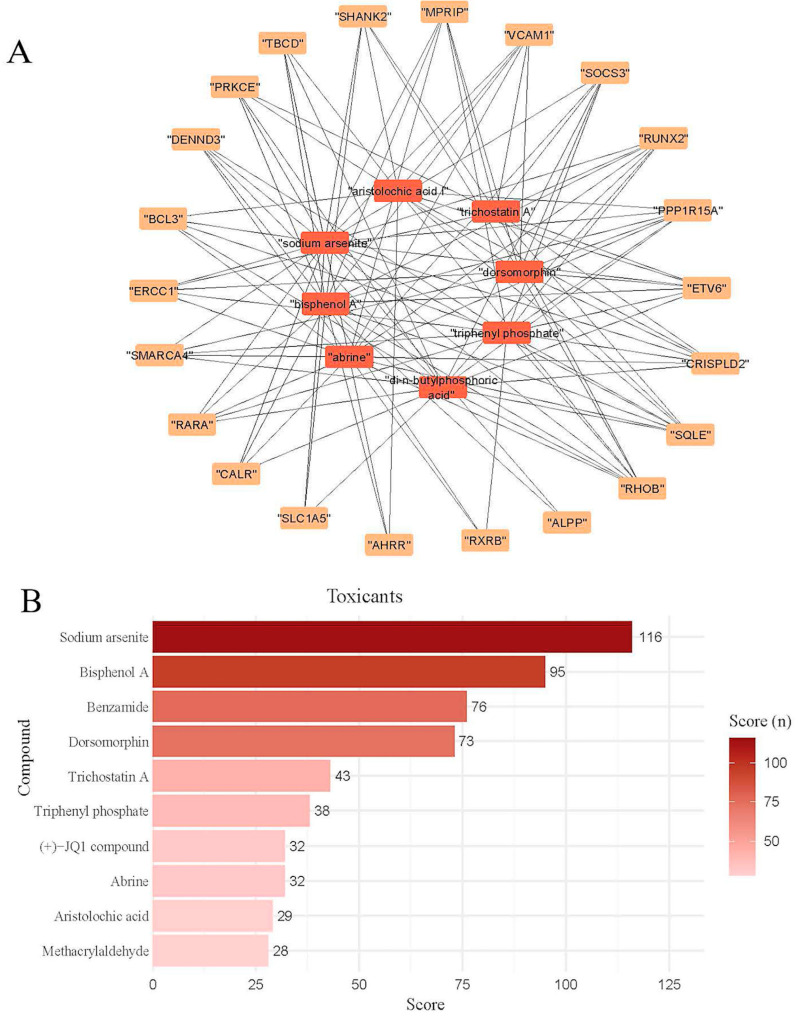
Network approach to chemical compounds (toxicants), their gene targets, and the top 10 chemical compounds most connected to all frailty-related CpG genes across cohorts. (**A**) Toxicological network plot. (**B**) Top 10 toxicants. Here, we have two types of nodes: biological targets (genes/proteins) and compounds (drugs and toxicants). In this scheme, color labels using a palette of pink-to-red shades represent toxicants. On the other hand, the edges (vertices) represent interactions or relationships among compounds and their targets. Each biological network shows the interactions (edges) between compounds (drugs or toxicants) and their gene targets. The intensity of the color of the nodes represents the number of connections (high degree centrality), meaning that with an intensely colored node, we have higher degree centrality.

Since it has been suggested that frailty may result from many environmental factors, including the chemical exposome, we performed a toxicological network analysis to understand the interactions between toxicants and the frailty-related genes. The network results reveal that at least 30 toxicants interact with the most frailty-related genes. Interestingly, the most common toxicants interacting with such genes are: sodium arsenite, bisphenol A, benzamide, dorsomorphin, trichostatin A, triphenyl phosphate, (+)-JQ1 compound, abrine, aristolochic acid, and metacrylaldehyde (Figure 5A).

**Figure 6:**
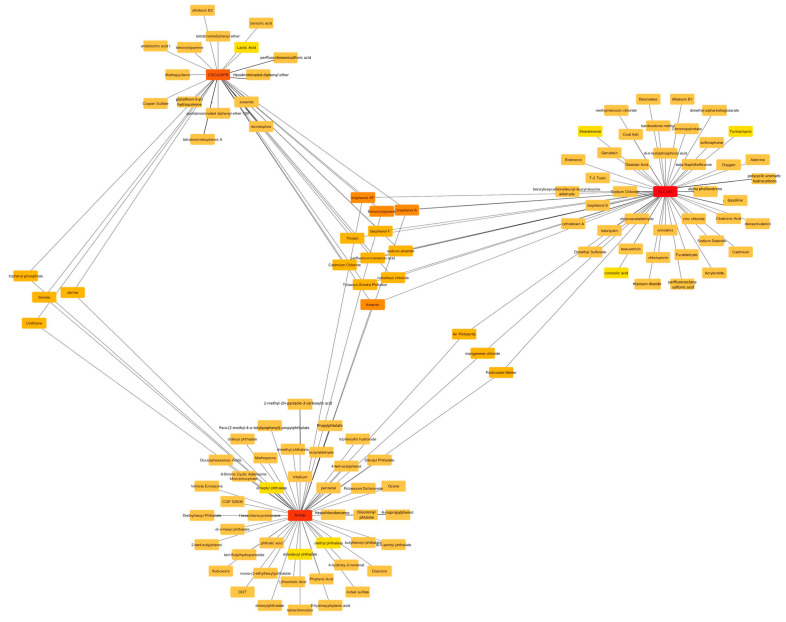
Toxicological network analysis reveals that 10 toxicants are shared by the genes RXRB, CDC42BPB, and SLC1A5. This figure depicts the interactions between the four frailty-related genes (red) and toxicants (color-coded from yellow to orange; less connected are colored in yellow, and more connected in orange). On the other hand, the edges represent interactions or relationships among compounds and their targets (genes).

Additionally, we aimed to determine whether the four shared frailty-related genes across cohorts were affected by the same toxicants. As seen in Figure 6, RXRB, CDC42BPB, and SLC1A5 have in common bisphenol A, particulate matter, air pollutants, abrine, triphenyl phosphate, smoke, arsenic, bisphenol AF, and benzo(α)pyrene. Meanwhile, CDC42BPB and SLC1A5 genes shared sodium arsenite, cadmium chloride, thiram, tobacco smoke pollution, bisphenol F, perfluoro-n-nonanoic acid, cobaltous chloride, and coumarin. These results suggest that exposure to such toxicants may modulate the DNAm patterns associated with frailty-related genes. Thus, the chemical exposome may be considered a potential factor responsible not only for the development of frailty but also for heterogeneity in manifestations among individuals with this syndrome. 

## 4. Discussion

### 4.1. Summary of Main Findings

Frailty syndrome is highly prevalent in older adults, and it is associated with adverse outcomes such as higher mortality, increased risk of hospitalizations, and propensity to use long-term care assistance. Interestingly, the lack of consensus on the frailty diagnosis and limited understanding of its key biological mechanisms contribute to poor diagnostic accuracy, inadequate management of frailty, and low responsiveness to interventions. To overcome such limitations and improve the quality of life of frail older adults, epigenetics has attracted interest as a potential tool for elucidating the biological mechanisms underlying this pathology. In fact, there is a clear association between frailty and changes in DNAm patterns, supporting the view that CpG patterns may be considered potential biomarkers for this entity [5]. In this context, although several studies report CpG sites that are differentially methylated in patients with frailty, these studies are limited by their reliance on a single ethnic group [22], leaving a gap in the knowledge about the role of DNAm patterns in frail individuals from underrepresented ethnicities. Moreover, several studies report that environmental exposure plays a crucial role in shaping DNAm patterns [23]; however, it remains unclear whether the exposome is involved in the development of frailty. Consequently, to understand if DNAm patterns are similar between populations and understand the role of the exposome in the frailty heterogeneity, we performed a meta-analysis of proportions and of weighted methylated beta (β) using a random-effects model, based on data from eight cohorts from German, Scottish, British, Chinese, Canadian, Danish, and Swedish populations. Additionally, using data extracted from these studies, we performed a toxicological network analysis to identify which toxicants interact with the frailty-related genes epigenetically modulated by the CpGs used in the meta-analysis. 

In this context, our meta-analysis revealed a pooled prevalence of frailty of 17.4%, with substantial statistical heterogeneity. This estimate is lower than the prevalence reported globally using the deficit-accumulation approach, which corresponds to 24% [24]. Similarly, our meta-analysis results comparing weighted methylation β also support this variability. Even after accounting for the use of the same frailty index across cohorts, these results suggest that further studies need to increase sample size, include underrepresented ethnicities, and, most importantly, account for distinct environmental exposures (the chemical exposome) experienced by each population. 

### 4.2. Epigenetic Signatures of Frailty and Shared Molecular Pathways Across Populations

To identify whether any CpGs are shared among the cohorts, we retrieved all CpGs reported by the studies in the meta-analysis and then performed a structural network analysis. The results demonstrate that the CpG site cg04772644 is common across the studies by Jiang M. [18] (Chinese cohort) and Li Xiangwei [17] (German cohort), suggesting that particular DNAm patterns in each cohort may be attributable to exposure to socioeconomic, biological, or environmental factors. To test this hypothesis, we annotated all genes corresponding to the listed CpGs and constructed a structural network to identify which genes are shared across cohorts. The results from this analysis demonstrated that four genes (CDC42BPB, SLC1A5, RXRB, and SLC22A18AS) were shared across the German [17], Chinese [18], Swedish, and Danish [22] cohorts. Interestingly, the *RXRB* gene corresponds to the cg04772644 CpG shared across both the German and the Chinese cohorts; in addition, this nuclear receptor in the X retinoid receptor family modulates gene transcription in response to retinoic acid [25]. These pathways point to alterations in vitamin D signaling and cellular metabolism, reinforcing the biological coherence of the shared epigenetic signals despite population differences. These findings are consistent with previous research showing that vitamin D plays a crucial role in metabolic pathways associated with frailty [26]. 

Regarding the other shared genes, the functional enrichment analysis (via TopGO) using *CDC42BPB* (CDC42 binding protein kinase beta, with a predominant role in regulating actin cytoskeleton and cellular polarity [27]), *SLC1A5* (Solute Carrier Family 1 Member 5, in charge of carrying neutral amino acids through the cellular membrane [28]), and *SLC22A18AS* (Solute Carrier Family 22 Member 18 Antisense, encodes a long non-coding RNA, involved in epigenetic regulation of the gene SLC22A18) revealed highly significant biological processes (adjusted *p* = 0.0043, fold enrichment = 100), including: L-glutamine import across the plasma membrane (GO:1903803), positive regulation of the vitamin D receptor signaling pathway (GO:0070564), L-aspartate import across the plasma membrane (GO:0140409), L-serine transport (GO:0015825), and glutamine transport (GO:0006868). These biological processes have implications for sarcopenia (loss of muscle mass, strength, and physical performance), which is a cornerstone of frailty, since glutamine is the most abundant amino acid in the blood and muscle. Hence, low bioavailability of this amino acid is associated with protein degradation and, ultimately, muscle loss [29]. Furthermore, L-aspartate, as a membrane transporter in the malate–aspartate shuttle, also has direct implications for sarcopenia, as dysfunction of this transporter may diminish ATP production in mitochondrial muscle cells, driving the organism to a state of energetic imbalance in skeletal muscle [30].

Additionally, in the functional enrichment analysis, two main KEGG pathways were identified: the thrombin signaling through protease-activated receptors (PARs) pathway and the biochemical process related to the Gα (12/13) signaling cascade. Meanwhile, the GO-BP enrichment analysis showed enrichment in T-cell differentiation, the retinoic acid receptor signaling pathway, and alpha–beta T-cell differentiation. These results align with previous genome-wide association studies (GWAS) that have linked inflammatory pathways, nuclear factor-κB (NFκB)-mediated protein kinase signaling cascades, and an epigenetic regulatory mechanism encoded by the METTL16 gene [31]. Similarly, our results align with a GWAS performed by Foote et al. [32], which found that the G protein-coupled receptor signaling cascade, as well as related mechanisms that maintain cellular function by regulating cell–cell adhesion, cellular migration, and cytoskeletal maintenance [29], was highly enriched in frailty. In addition, our study suggests that frailty may be associated with alterations in thrombin signaling through the PAR pathway, making it, to our knowledge, the first to report these results, given the consistent link between frailty and immune system pathways. In this context it is well known that such pathways have multiple implications on the immune system such as thrombin-activated platelets, the endothelium and immune cells through the PARs, which amplify the immune response in an individual, releasing pro-inflammatory cytokines, increasing the gene expression of adhesion molecules and promoting a state of oxidative stress; this whole biological process contributes to a multisystemic decline prototypical in individuals with frailty [33]. 

### 4.3. The Chemical Exposome as a Contributor to Frailty Heterogeneity

Exposure to air pollutants, such as tobacco smoke and solid fuels, has been reported to be associated with an increased risk of frailty [34]. In this regard, we conducted a toxicological network analysis to examine the role of environmental exposure in the development of frailty. According to our main findings, toxicants such as bisphenol A, sodium arsenite, arsenic, bisphenol AF, benzo(α) pyrene, cadmium chloride, thiram, tobacco smoke pollution, bisphenol F, perfluoro-n-nonanoic acid, and cobaltous chloride interact with three of the frailty-related genes (SLC1A5, CDC42BPB, and RXRB) as is shown in Figure 6. This evidence aligns with previous studies, which demonstrate a significant correlation between exposure to heavy metal mixtures and the onset of frailty in middle-aged and older adults [35]. Additionally, our results suggest that bisphenol A may be associated with frailty. Interestingly, endocrine disruptors, such as phthalates and phenols, have been reported to be strongly associated with higher odds of developing frailty (e.g., bisphenol A; odds ratio (OR) = 1.21; 95% confidence interval (CI), 1.04–1.40) [36]. Apart from these results, to our knowledge, this is the first report linking sodium arsenite to frailty syndrome, suggesting that studies on the relationship between heavy metals and frailty are needed to understand this correlation and its possible mechanisms. 

### 4.4. Strengths and Limitations

This study has several strengths. First, to our knowledge, it is the first systematic review and meta-analysis that integrates epigenetic data with network toxicology analysis to explore the role of the chemical exposome in frailty. Second, we included cohorts from different ethnic backgrounds (German [17], Chinese [18], Swedish, and Danish [22] cohorts), allowing us to identify consistent epigenetic signals across populations despite high heterogeneity. Third, the study was prospectively registered with PROSPERO and conducted in accordance with the PRISMA 2020 guidelines, enhancing transparency and reproducibility. Additionally, we performed sensitivity analyses and used both prevalence and weighted methylation beta meta-analyses, providing a more comprehensive assessment of the epigenetic landscape of frailty.

However, some limitations should be acknowledged. The high statistical heterogeneity observed in both prevalence and methylation beta estimates limits the generalizability of our findings. This heterogeneity likely reflects differences in study populations. Furthermore, the number of eligible studies was relatively small, and only five provided sufficient data for the meta-analysis of methylation beta values. Moreover, there are significant sample imbalance in the studies included in the analysis, which contributes to the heterogeneity previously mentioned; hence, we performed weighted methylated beta meta-analysis to have a more consistent comparison parameter. Unfortunately, this analysis only includes five out of eight studies as these were the only studies to report the false discovery rate (FDR), *p*-value, and standard error (S.E) for both hypo- and hypermethylated CpGs.

Most included studies were conducted in European and East Asian populations, which restricts the extrapolation of results to other ethnic groups, particularly those from Latin America and Africa. In addition, because this is an observational study using aggregated data, causal inferences cannot be drawn regarding the relationship between the chemical exposome and frailty-related epigenetic changes. Finally, individual-level exposure data were unavailable, limiting our ability to directly link specific environmental chemicals to DNA methylation alterations in frail individuals. Finally, our conclusions are limited to our study and should be interpreted with caution, as our results require experimental validation and have not been tested across other ethnicities.

## 5. Conclusions and Future Perspectives

In summary, frailty is a highly heterogeneous clinical syndrome, with variability in prevalence and epigenetic signatures across populations strongly suggesting that it is modulated by the chemical exposome. Despite this heterogeneity, our meta-analysis found that frailty is predominantly characterized by a hypomethylated profile; only the CpG site (cg04772644) is shared among the available studies. Additionally, four frailty-related genes (CDC42BPB, SLC1A5, RXRB, and SLC22A18AS) were shared across diverse ethnic groups. Finally, by integrating enrichment analyses and network toxicology, this study provides a novel framework linking environmental exposures to the epigenetic architecture of frailty. These findings highlight the importance of exposome-wide approaches for advancing precision prevention and management of frailty in aging populations. 

This study compiles the epigenetic evidence in relation to frailty. We conducted toxicology network analysis to establish the association of this syndrome with factors embedded in the environment. However, it is necessary to develop experimental studies to further explore the potential causal relationship between chemical compounds and frailty. Furthermore, we hope this study will develop interest in conducting epigenome-wide association studies in other ethnicities that are yet to be studied.

## Figures and Tables

**Table 2:** Pathway and functional enrichment analysis of genes identified through the interactome analysis of frailty-related genes, as per the CTD. The table includes results from reactome pathway analysis (upper) and GO terms from a biological process analysis (lower).

Pathway Enrichment Analysis with ReactomePA					
Enriched Pathways	Pathway ID	Adjusted *p*-Value	Fold Enrichment	Gene ID	CpG Sites
Thrombin signaling through proteinase-activated receptors (PARs)	R-HSA-456926	0.0138	37.3191	F2RL3, GNB1, GNG12	cg04987734, cg26568075, cg03636183, cg25189904
G alpha (z) signaling events	R-HSA-418597	0.0234	24.8794	GNB1, GNG12, PRKCE	cg26568075, cg03636183, cg25189904, cg25453797
Platelet activation, signaling, and aggregation	R-HSA-76002	0.0241	7.5678	F2RL3, GNB1, GNG12, PRKCE, RHOB	cg04987734, cg26568075, cg03636183, cg25189904, cg25453797, cg13471188
G-beta:gamma signaling through CDC42	R-HSA-8964315	0.0241	44.2301	GNB1, GNG12	cg26568075, cg03636183, cg25189904
Prostacyclin signaling through prostacyclin receptor	R-HSA-392851	0.0241	41.9022	GNB1, GNG12	cg26568075, cg03636183, cg25189904
**Enrichment Analysis with TopGO**					
GO Terms	GO ID	Adjusted *p*-value	Fold Enrichment		
T-cell differentiation	GO:0030217	0.0212	10.4702		
Retinoic acid receptor signaling pathway	GO:0048384	0.0212	47.8638		
Alpha–beta T-cell differentiation	GO:0046632	0.0312	16.7943		
Lymphocyte differentiation	GO:0030098	0.0312	7.8649		
Endochondral bone morphogenesis	GO:0060350	0.0375	27.9205		

**Table 3:** Functional enrichment analysis of the four shared genes was performed. The table includes GO terms from a biological process analysis.

Enrichment Analysis with TopGO			
GO Terms	GO ID	Adjusted *p*-Value	Fold Enrichment
L-glutamine import across the plasma membrane	GO:1903803	0.0043	100
Positive regulation of the vitamin D receptor signaling pathway	GO:0070564	0.0043	100
L-aspartate import across the plasma membrane	GO:0140409	0.0043	100
L-serine transport	GO:0015825	0.0043	100
Glutamine transport	GO:0006868	0.0043	100

## Data Availability

No new data were created or analyzed in this study. Data sharing is not applicable to this article.

## Supplementary Materials

The following supporting information can be downloaded at: https://www.mdpi.com/article/10.3390/ijms27135986/s1.

## Abbreviations

CI	Confidence interval
CpG	Cytosine-phosphate-guanine dinucleotide
CTD	Comparative Toxicogenomics Database
DNAm	DNA methylation
FDR	False discovery rate
FI	Frailty index
GO	Gene Ontology
GWAS	Genome-wide association study
HGNC	HUGO Gene Nomenclature Committee
JBI	Joanna Briggs Institute
KEGG	Kyoto Encyclopedia of Genes and Genomes
MCC	Maximum Neighborhood Component
MeSH	Medical Subject Headings
NF-κB	Nuclear factor kappa-light-chain-enhancer of activated B cells
OR	Odds Ratio
PARs	Protease-activated receptors
PICO	Population, Intervention, Comparison, Outcome
PRISMA	Preferred Reporting Items for Systematic Reviews and Meta-Analyses
PROSPERO	International Prospective Register of Systematic Reviews
SE	Standard error
β	Beta coefficient

## References

[1] Cesari M., Marzetti E., Canevelli M., Guaraldi G. (2017). Geriatric Syndromes: How to Treat. Virulence.

[2] Dong Y., Hu Q. (2025). Association between Frailty and Frailty Change with Chronic Lung Disease: Results from Two Prospective Cohort Studies. BMC Public Health.

[3] Garcia-Gimenez J.L. (2021). Epigenetics in Precision Medicine.

[4] Gao X., Zhang Y., Saum K.-U., Schöttker B., Breitling L.P., Brenner H. (2017). Tobacco Smoking and Smoking-Related DNA Methylation Are Associated with the Development of Frailty among Older Adults. Epigenetics.

[5] Lozupone M., Solfrizzi V., Sardone R., Dibello V., Castellana F., Zupo R., Lampignano L., Bortone I., Daniele A., Panza F. (2024). The Epigenetics of Frailty. Epigenomics.

[6] Richardson B. (2003). Impact of Aging on DNA Methylation. Ageing Res. Rev..

[7] Tay J.H., Barros D., Wang W., Wazny V.K., Maier A.B. (2025). Biological Age Measured by DNA Methylation Clocks and Frailty: A Systematic Review and Meta-Analysis. Lancet Healthy Longev..

[8] Feil R., Fraga M.F. (2012). Epigenetics and the Environment: Emerging Patterns and Implications. Nat. Rev. Genet..

[9] Page M.J., McKenzie J.E., Bossuyt P.M., Boutron I., Hoffmann T.C., Mulrow C.D., Shamseer L., Tetzlaff J.M., Akl E.A., Brennan S.E. (2021). The PRISMA 2020 Statement: An Updated Guideline for Reporting Systematic Reviews. BMJ.

[10] Munn Z., Moola S., Lisy K., Riitano D., Tufanaru C. (2015). Methodological Guidance for Systematic Reviews of Observational Epidemiological Studies Reporting Prevalence and Cumulative Incidence Data. Int. J. Evid. Based Healthc..

[11] Viechtbauer W. (2010). Conducting Meta-Analyses in R with the metafor Package. J. Stat. Softw..

[12] Li X., Delerue T., Schöttker B., Holleczek B., Grill E., Peters A., Waldenberger M., Thorand B., Brenner H. (2022). Derivation and Validation of an Epigenetic Frailty Risk Score in Population-Based Cohorts of Older Adults. Nat. Commun..

[13] Yu G., He Q.-Y. (2016). ReactomePA: An R/Bioconductor Package for Reactome Pathway Analysis and Visualization. Mol. Biosyst..

[14] Davis A.P., Wiegers T.C., Sciaky D., Barkalow F., Strong M., Wyatt B., Wiegers J., McMorran R., Abrar S., Mattingly C.J. (2025). Comparative Toxicogenomics Database’s 20th Anniversary: Update 2025. Nucleic Acids Res..

[15] Dehmer M., Emmert-Streib F., Graber A., Salvador A. (2011). Applied Statistics for Network Biology: Methods in Systems Biology.

[16] Chin C.-H., Chen S.-H., Wu H.-H., Ho C.-W., Ko M.-T., Lin C.-Y. (2014). cytoHubba: Identifying Hub Objects and Sub-Networks from Complex Interactome. BMC Syst. Biol..

[17] Zhang Y., Saum K.-U., Schöttker B., Holleczek B., Brenner H. (2018). Methylomic Survival Predictors, Frailty, and Mortality. Aging.

[18] Jiang M., Guo P., Liu S., Wang H., Cai J., Qin J., Gao X. (2025). A Multidimensional Epidemiological Perspective on Frailty Dynamics and Prevention. Trends Endocrinol. Metab..

[19] Gale C.R., Marioni R.E., Harris S.E., Starr J.M., Deary I.J. (2018). DNA methylation and the epigenetic clock in relation to physical frailty in older people: The Lothian Birth Cohort 1936. Clin. Epigenet..

[20] Collerton J., Gautrey H.E., van Otterdijk S.D., Davies K., Martin-Ruiz C., von Zglinicki T., Kirkwood T.B., Jagger C., Mathers J.C., Strathdee G. (2014). Acquisition of aberrant DNA methylation is associated with frailty in the very old: Findings from the Newcastle 85+ Study. Biogerontology.

[21] Chadha S. Identification of DNA Methylation Signature of Frailty in Postmenopausal Women and Extracellular Vesicle Mediated Epigenetic Age Reversal in Skeletal Myoblasts. http://hdl.handle.net/1993/37660.

[22] Mak J.K.L., Skovgaard A.C., Nygaard M., Kananen L., Reynolds C.A., Wang Y., Kuja-Halkola R., Karlsson I.K., Pedersen N.L., Hägg S. (2024). Epigenome-Wide Analysis of Frailty: Results from Two European Twin Cohorts. Aging Cell.

[23] Watkins S.H., Testa C., Chen J.T., De Vivo I., Simpkin A.J., Tilling K., Diez Roux A.V., Davey Smith G., Waterman P.D., Suderman M. (2023). Epigenetic Clocks and Research Implications of the Lack of Data on Whom They Have Been Developed: A Review of Reported and Missing Sociodemographic Characteristics. Environ. Epigenet..

[24] O’Caoimh R., Sezgin D., O’Donovan M.R., Molloy D.W., Clegg A., Rockwood K., Liew A. (2021). Prevalence of Frailty in 62 Countries across the World: A Systematic Review and Meta-Analysis of Population-Level Studies. Age Ageing.

[25] Szanto A., Narkar V., Shen Q., Uray I.P., Davies P.J.A., Nagy L. (2004). Retinoid X Receptors: X-Ploring Their (patho)physiological Functions. Cell Death Differ..

[26] Mishra M., Wu J., Kane A.E., Howlett S.E. (2024). The Intersection of Frailty and Metabolism. Cell Metab..

[27] Moncrieff C.L., Bailey M.E., Morrison N., Johnson K.J. (1999). Cloning and Chromosomal Localization of Human Cdc42-Binding Protein Kinase Beta. Genomics.

[28] Freidman N., Chen I., Wu Q., Briot C., Holst J., Font J., Vandenberg R., Ryan R. (2020). Amino Acid Transporters and Exchangers from the SLC1A Family: Structure, Mechanism and Roles in Physiology and Cancer. Neurochem. Res..

[29] Meynial-Denis D. (2016). Glutamine Metabolism in Advanced Age. Nutr. Rev..

[30] Yin M., Zhang H., Liu Q., Ding F., Hou L., Deng Y., Cui T., Han Y., Chen Y., Huang C. (2022). Determination of Skeletal Muscle Mass by Aspartate Aminotransferase / Alanine Aminotransferase Ratio, Insulin and FSH in Chinese Women with Sarcopenia. BMC Geriatr..

[31] Ye Y., Noche R.B., Szejko N., Both C.P., Acosta J.N., Leasure A.C., Brown S.C., Sheth K.N., Gill T.M., Zhao H. (2023). A Genome-Wide Association Study of Frailty Identifies Significant Genetic Correlation with Neuropsychiatric, Cardiovascular, and Inflammation Pathways. Geroscience.

[32] Foote I.F., Flint J.P., Fürtjes A.E., Lawrence J.M., Mullin D.S., Fisk J.D., Karakach T.K., Rutenberg A., Martin N.G., Lupton M.K. (2025). Uncovering the Multivariate Genetic Architecture of Frailty with Genomic Structural Equation Modeling. Nat. Genet..

[33] Walston J., McBurnie M.A., Newman A., Tracy R.P., Kop W.J., Hirsch C.H., Gottdiener J., Fried L.P. (2002). Cardiovascular Health Study Frailty and Activation of the Inflammation and Coagulation Systems with and without Clinical Comorbidities: Results from the Cardiovascular Health Study. Arch. Intern. Med..

[34] Ding Q., Kou C., Feng Y., Sun Z., Geng X., Sun X., Jia T., Wang Q., Huang Q., Han W. (2024). Effects of Air Pollutants Exposure on Frailty Risk: A Systematic Review and Meta-Analysis. Environ. Pollut..

[35] Teixeira-Gomes A., Lage B., Esteves F., Sousa A.C., Pastorinho M.R., Valdiglesias V., Costa S., Laffon B., Teixeira J.P. (2020). Frailty Syndrome, Biomarkers and Environmental Factors—A Pilot Study. Toxicol. Lett..

[36] Del Río Barrera T., Zambrano Ledesma K.N., Aguilar Hernández M., Reyes Chávez K., Aguirre Barajas A.F., Alvarez Vázquez D.P., Garcia Santiago G., Arias Castro A. (2025). Endocrine Disruptors and Their Impact on Quality of Life: A Literature Review. Cureus.

