# Supplementary figures and images for: Epigenetic Signatures of Frailty: A Systematic Review, Meta-Analysis, and Network Analysis of the Chemical Exposome
Source: Int J Mol Sci. 2026 Jul 3;27(13):5986. doi: 10.3390/ijms27135986 (PMC13361453; doi:10.3390/ijms27135986)

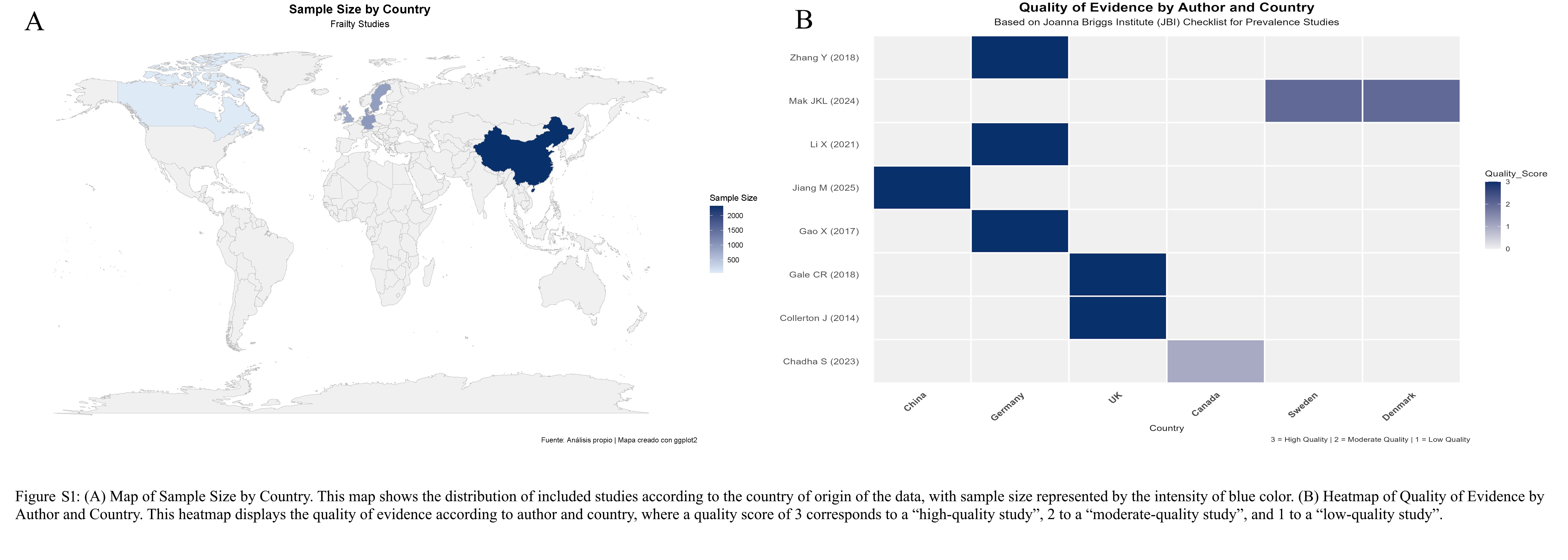

Supplement: Supplementary file 1 [file ijms-27-05986-s001.zip › Figure S1.jpg]

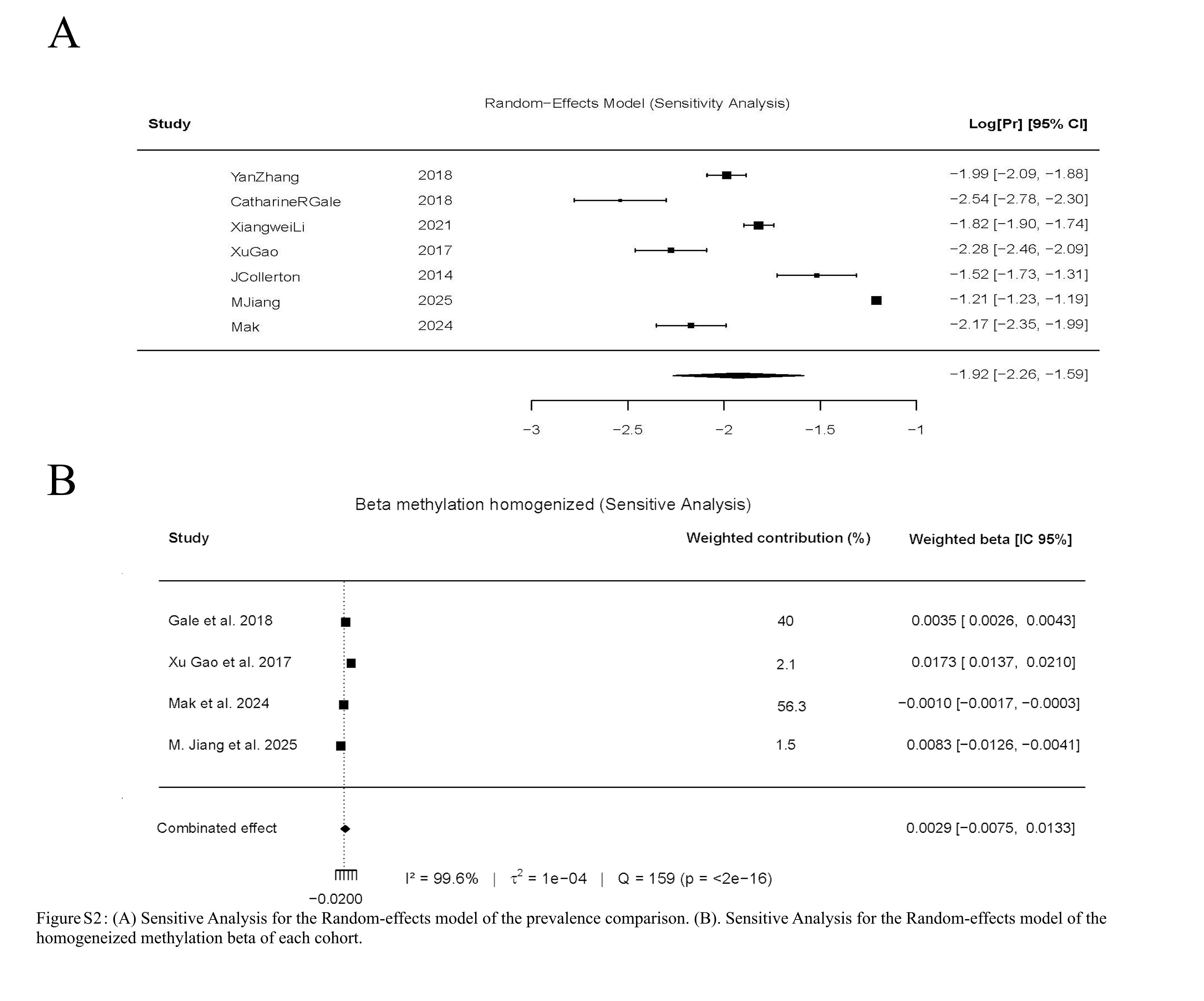

Supplement: Supplementary file 1 [file ijms-27-05986-s001.zip › Figure S2.jpg]
